# Whey Protein Isolate/Calcium Silicate Hydrogels for Bone Tissue Engineering Applications—Preliminary In Vitro Evaluation

**DOI:** 10.3390/ma16196484

**Published:** 2023-09-29

**Authors:** Tayla Ivory-Cousins, Aleksandra Nurzynska, Katarzyna Klimek, Daniel K. Baines, Wieslaw Truszkiewicz, Krzysztof Pałka, Timothy E. L. Douglas

**Affiliations:** 1 School of Engineering, Faculty of Mechanical Engineering, Lancaster University, Nadbystrzycka 36 Street, Gillow Avenue, Lancaster LA1 4YW, UK; taylaivory1999@googlemail.com (T.I.-C.); d.baines3@lancaster.ac.uk (D.K.B.); 2 Chair and Department of Biochemistry and Biotechnology, Medical University of Lublin, Chodzki 1 Street, 20-093 Lublin, Poland; aleksandra.nurzynska@umlub.pl (A.N.); katarzyna.klimek@umlub.pl (K.K.); wieslaw.truszkiewicz@umlub.pl (W.T.); 3 Faculty of Mechanical Engineering, Lublin University of Technology, Nadbystrzycka 36 Street, 20-618 Lublin, Poland; k.palka@pollub.pl

**Keywords:** whey protein, calcium silicate, bone scaffolds, SEM, FTIR, swelling, osteoblasts, cytocompatibility

## Abstract

Whey protein isolate (WPI) hydrogels are attractive biomaterials for application in bone repair and regeneration. However, their main limitation is low mechanical strength. Therefore, to improve these properties, the incorporation of ceramic phases into hydrogel matrices is currently being performed. In this study, novel whey protein isolate/calcium silicate (WPI/CaSiO_3_) hydrogel biomaterials were prepared with varying concentrations of a ceramic phase (CaSiO_3_). The aim of this study was to investigate the effect of the introduction of CaSiO_3_ to a WPI hydrogel matrix on its physicochemical, mechanical, and biological properties. Our Fourier Transform Infrared Spectroscopy results showed that CaSiO_3_ was successfully incorporated into the WPI hydrogel matrix to create composite biomaterials. Swelling tests indicated that the addition of 5% (*w*/*v*) CaSiO_3_ caused greater swelling compared to biomaterials without CaSiO_3_ and ultimate compressive strength and strain at break. Cell culture experiments demonstrated that WPI hydrogel biomaterials enriched with CaSiO_3_ demonstrated superior cytocompatibility in vitro compared to the control hydrogel biomaterials without CaSiO_3_. Thus, this study revealed that the addition of CaSiO_3_ to WPI-based hydrogel biomaterials renders them more promising for bone tissue engineering applications.

## 1. Introduction

Hydrogels are water-swollen polymeric materials that have a similar porous structure to the extracellular matrix (ECM). Moreover, they easily facilitate the transport of nutrients and they are also considered non-toxic towards cells [1,2,3,4]. Thanks to these advantageous properties, many naturally derived (e.g., carbohydrate-based, protein-based) and synthetically derived hydrogels are used in various biomedical applications, including skin tissue engineering (STE), nerve tissue engineering (NTE), cartilage tissue engineering (CTE), and bone tissue engineering (BTE) [3,5,6,7,8].

Among the protein-based hydrogels, those composed of whey proteins seem especially interesting for BTE applications. Whey proteins are a waste product in the dairy industry (from cheese manufacturing), which makes them inexpensive, available in copious amounts, and easily accessible. There are many forms of whey protein, such as reduced-lactose whey, demineralized whey, whey protein concentrates, and whey protein isolates [9,10]. The composition of whey protein consists of mainly β-lactoglobulin (β-Lg); α-lactalbumin (α-La); and small amounts of glycomacropeptide (GMP), immunoglobulins (Igs), bovine serum albumin (BSA), lactoferrin (LF), lactoperoxidase (LP), and proteose peptone (PP) [11].

Whey protein isolate (WPI) contains at least 90% protein and its main component (β-Lg) can enhance human immune responses and acts as an antioxidant, antitumor, antiviral, and antibacterial agent [9,10]. By showing that cell proliferation was not stimulated in β-Lg-depleted milk, Tai, Chen, and Chen proved that β-Lg is the major protein in bovine milk that stimulates cell proliferation [12].

The investigation of the influence of WPI on different human cells is the first step in assessing its potential in BTE. Douglas et al. [9] tested the effect of different WPI concentrations on the response of human osteoblast-like Saos-2 cells, human adipose tissue-derived stem cells (ADSCs), and human neonatal dermal fibroblasts (FIB). From this study, in which cell adhesion and proliferation, osteogenic differentiation, and calcium deposition were evaluated and quantified, many positive results were achieved. With promising conclusions drawn on all three cell types, the most positive effects of WPI were seen on the proliferation of Saos-2 osteoblast-like cells and fibroblasts and the osteogenic differentiation of ADSCs. On the basis of this research, WPI was considered a promising component for hydrogels, specifically for bone tissue regeneration, and hence, subsequent research was performed. Moreover, to form a WPI hydrogel, no chemical crosslinking agents are required. This makes it very attractive for biomedical applications. The heat treatment of an aqueous solution of whey protein isolate (above 60 °C) results in unfolding of the proteins followed by the formation of interprotein bonds, leading to the formation of a three-dimensional network filled with water, i.e., a WPI hydrogel [11]. Nevertheless, despite their high cytocompatibility, WPI hydrogels possessed relatively low mechanical properties in the context of BTE applications compared to ceramic biomaterials such as calcium phosphate (CaP). These might be improved by the introduction of ceramic phases. An example is a WPI/gelatin/CaP composite hydrogel prepared by Dziadek et al. [11]. This composite hydrogel aimed to improve the mechanical properties and biological features of WPI hydrogels for application in BTE. A combination of two techniques was used to produce this hydrogel. The first included a combination of various materials to obtain multicomponent hydrogels. The second involved modification of the hydrogel matrix with ceramic particles. This led to the hydrogel consisting of WPI as the main hydrogel matrix component (for the first time), gelatin as a matrix modifier, and alpha-tricalcium phosphate (α-TCP) as a ceramic filler. It was discovered from this research that increasing the α-TCP concentration linearly improved the mechanical properties of the composite in comparison to the control hydrogels.

Another example is WPI and aragonite composite hydrogel biomaterials. Aragonite is a form of calcium carbonate, which occurs naturally in marine coral. The preparation of these WPI/aragonite hydrogels involved the incorporation of synthetic aragonite rod-like powder in three different concentrations (100 mg/mL, 200 mg/mL, and 300 mg/mL). It was found that as the concentration of aragonite increased, the composites swelled more and released more protein under physiologically relevant degradation conditions. Mechanical strength did increase due to the addition of aragonite; however, this was not a linear trend; the increase was only significant for the 300 mg/mL hydrogel composite [10].

Taking into account the aforementioned results associated with the use of CaP and aragonite, it was decided to develop WPI hydrogel biomaterials enriched with another ceramic phase, namely, calcium silicate (CaSiO_3_). CaSiO_3_ bioceramics were used in this study due to their beneficial properties in the context of bone tissue as both calcium (Ca) and silicon (Si) ions being involved in many biological processes. Ca is essential in bone growth and blood vessels, and moreover, favors osteoblast proliferation, differentiation, and ECM mineralization. Si is extremely important in the calcification of bone, specifically in the metabolic process. Furthermore, Si can help to enhance bone density and inhibit osteoporosis [13,14,15,16]. In vitro cell culture studies have shown that CaSi-based materials can support the attachment, proliferation, and differentiation of human bone mesenchymal stem cells (hMSC). Zhang et al. tested pseudowollastonite (a high-temperature polymorph of β-CaSiO_3_) due to its osteoconductive nature. They examined coarse- and fine-grained surfaces of β-CaSiO_3_ and showed their beneficial effects on the adhesion, viability, proliferation, and differentiation of hMSC [17]. Therefore, we hypothesize that the addition of wollastonite, a metasilicate composed of naturally occurring CaSiO_3_, should improve the mechanical properties of WPI hydrogel and promote osteoblast proliferation and differentiation, which, in turn, may lead us to obtain a very promising scaffold for bone tissue engineering applications. Hence, the aim of this study was to fabricate WPI-based hydrogel biomaterials enriched with CaSiO_3_ and assess their structural, physicochemical, mechanical, and biological properties. Another aim of this work was to evaluate the biomedical potential of the fabricated biomaterials as future scaffolds for BTE.

## 2. Materials and Methods

### 2.1. Preparation of WPI-Based Biocomposites

First, two different suspensions of CaSiO_3_ in distilled water were prepared in order to obtain a concentration of CaSiO_3_ equal to 2.32% or 5%. Then, a 40% (*w*/*v*) WPI solution was obtained by adding 10 g of WPI powder to 25 mL of distilled water for the control biomaterials or to 25 mL of the 2 different suspensions of CaSiO_3_. The mixtures were vortexed for 10 s and subjected to homogenization for 24 h (IKA Loopster homogenizer, IKA England LTD, Oxon, UK). Next, 1 mL of each biomaterial composition was pipetted into 2 mL Eppendorf tubes and the samples were placed in a water bath (set to 90 °C) for 20 min. The cross-linked biomaterials were then sterilized via autoclaving at 120 °C for 2 h. Therefore, the formed composite biomaterials were: 40% WPI/0% CaSiO_3_ (control), 40% WPI/2.32% CaSiO_3_, and 40% WPI/5% CaSiO_3_. The compositions of each biomaterial formed in this study and their symbols are presented in Table 1.

### 2.2. Scanning Electron Microscope (SEM) Observations

SEM observations were made using a Nova NanoSEM 450 microscope (FEI, Eindhoven, The Netherlands) in a low vacuum at an accelerating voltage of 5 kV. An LVD detector was used to perform image surface topography and a GAD detector for chemical composition differentiation.

### 2.3. Swelling Analysis

Swelling analysis of the biomaterials was performed by measuring their ability to absorb fluid over time. Autoclaved biomaterials of 40/0, 40/2.32, and 40/5 were incubated in solutions of phosphate-buffered saline (PBS) and simulated body fluid (SBF) for 7 days at 37 °C. The biomaterials (5 for each group) were weighed at 0 h, 24 h, 48 h, and 168 h. Before weighing, they were first placed on absorbent paper to remove solution present on the surface. The swelling ratio of each sample was calculated using Equation (1):

(1) $$\frac{WA-WB}{WB}\times 100$$

where WA denotes the weight after swelling, while WB denotes the weight before swelling. The PBS was produced by dissolving 1 phosphate-buffered saline tablet in 200 mL of distilled water to obtain a 137 mM NaCl, 2.7 mM KCl, and 10 mM phosphate-buffered saline, which had a pH of 7.4 at 25 °C. SBF was prepared according to the procedure described previously in detail in [18]. SBF also has a pH of 7.4 when adjusted using 1.0 mol of HCl after adding the other reagents. All reagents used for the preparation of PBS and SBF were supplied by Merck Life Science, Gillingham, Dorset, UK.

### 2.4. Mechanical Compression Testing

Compression testing was carried out using an Instron 3345 5 kN testing machine (Instron, Norwood, MA, USA) in a manner similar to that described in our previous publications [10,11]. Bluehill Universal software (Bluehill Universal|Instron) was used to achieve a displacement rate equal to 4 mm min^−1^ until sample failure. Biomaterials of diameter 8 mm and height 10 mm were compressed. The effect of both 40/0 and 40/2.32 as well as 40/5 on the mechanical properties were assessed (*n* = 10).

### 2.5. Fourier Transform Infrared (FTIR) Spectroscopy Analysis

Spectra in the mid-IR region (Agilent Technology, Cheadle, UK) were obtained (4000–650 cm^−1^) as this region corresponds to the majority of primary absorption frequencies [19]. The mid-IR spectrum is divided into 4 regions: the single-bond region (2500–4000 cm^−1^), the triple-bond region (2000–2500 cm^−1^), the double-bond region (1500–2000 cm^−1^), and the fingerprint region (600–1500 cm^−1^) [20]. An average of 32 scans with a resolution equivalent to 4 cm^−1^ were performed on each sample.

The dried biomaterials were analyzed using this technique. This was carried out so that the large, broad peaks that correspond to O-H were removed, allowing peaks corresponding to WPI and CaSiO_3_ to be visualized. CaSiO_3_ powder was also later analyzed using FTIR.

### 2.6. Cytocompatibility In Vitro

Cell culture experiments in vitro were performed using normal human fetal osteoblasts (hFOB 1.19 cell line, ATCC, Hertford, UK). The cells were cultured according to the ATCC instructions as described by us in detail previously [21]. The cell culture experiments included an evaluation of cell proliferation and osteogenic differentiation in direct contact with the biomaterials. As a control, cells cultured on polystyrene (PS) were included. For the proliferation assessment, hFOB 1.19 cells were seeded on biomaterials or on PS surfaces at a concentration of 1 × 10^5^ cells and incubated for 3 and 6 days. After each time point, a WST-8 assay (Cell counting Kit-8, Merck Life Science, Gillingham, Dorset, UK) was performed to determine optical density (OD), which is strictly correlated with the number of living metabolically active cells. Furthermore, after this step (WST-8 assay is non-cytotoxic for cells), osteoblasts were additionally fixed and stained with Hoechst 33342 (Merck Life Science, Gillingham, Dorset, UK) and AlexaFluor^TM^ 635 Phalloidin (ThermoFisher Scientific, Waltham, MA, USA) dyes to visualize cell nuclei and cytoskeletons, respectively. The cells were observed and photographed using a confocal laser scanning microscope (CLSM, Olympus Fluoview equipped with FV1000, Shinjuku, Japan). In order to evaluate the osteogenic differentiation of osteoblasts, cells were also seeded directly on the biomaterials or on the PS surfaces (control experiment) at a concentration of 1 × 10^5^ cells. After 24 h of incubation, the basic medium was replaced with an osteogenic one (basic medium supplemented with 10^−7^ M dexamethasone, 10 mM β-glycerophospate, and 0.05 mg/mL ascorbic acid; all reagents from Merck Life Science, Gillingham, Dorset, UK). After 7 and 21 days of incubation, the relative expression of osteogenic genes, namely, collagen I (Col I), bone alkaline phosphatase (bALP), and osteocalcin (OC), was evaluated via RT-qPCR analysis. The RT-qPCR procedure was performed according to the method described in detail in our previous work [22]. As a housekeeping gene, glyceraldehyde-3-phosphate dehydrogenase (GAPDH) was used. The list of primers, purchased from Merck Life Science, Gillingham, Dorset, UK, is presented in Table 2. The relative gene expressions were calculated via the 2^−∆∆Ct^ method according to the recommendations provided by Rao et al. [23]. All analyses were performed in three independent replicates using four biomaterials or PS (*n* = 4).

### 2.7. Statistical Analysis

The experiments were carried out in three independent runs using at least three biomaterial samples per experimental group. The results are displayed as mean values ± standard deviation (SD). First, the D’Agostino–Pearson omnibus normality test was applied to assess the normality of the distribution of the obtained data. Then, a One-Way ANOVA test was performed, followed by a Tukey’s multiple comparison test or Two-Way ANOVA test, followed by a Bonferroni comparison test to evaluate the statistical differences between the tested samples. When the *p*-value was lower than 0.05 (*p* < 0.05), differences were considered statistically significant (GraphPad Prism 5, Version 5.04, GraphPad Software, San Diego, CA, USA).

## 3. Results and Discussion

### 3.1. Surface Topography

**Figure 1:**
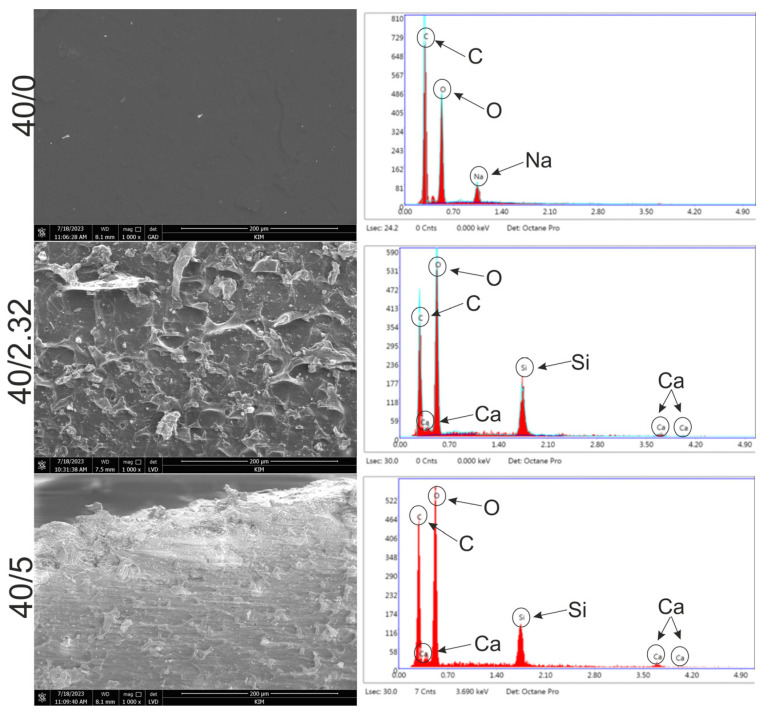
SEM images and EDS spectra of WPI-based biomaterials: 40/0 (control), 40/2.32, and 40/5.

The SEM observations revealed that the surface of the 40/0 biomaterials (control) possessed a smooth microstructure without any visible precipitates (Figure 1). The EDS spectrum showed that this biomaterial was composed of carbon, oxygen, and sodium, which confirms the lack of possibility of the occurrence of precipitates formed by metal salts. In turn, the microstructures of both the 40/2.32 and 40/5 biomaterials were rougher compared to those of the 40/0 biomaterials (Figure 1). In both cases, clusters of precipitates could be seen, which, as confirmed by the EDS analysis, most likely came from CaSiO_3_ powder.

### 3.2. Ability to Absorb Liquids

**Figure 2:**
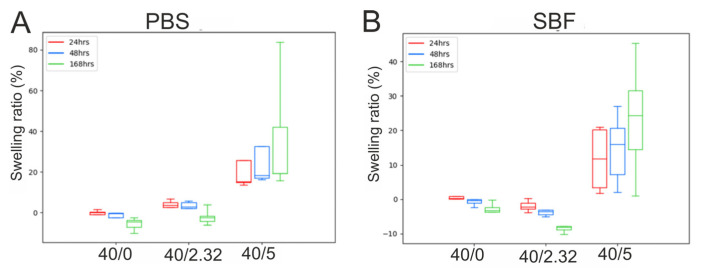
Ability of WPI-based biomaterials (40/0 (control), 40/2.23, and 40/5) to swell in contact with PBS (**A**) or SBF (**B**) after 24, 48, and 168 h of incubation.

Swelling testing in different media is used to evaluate hydrogel biomaterials’ ability to retain their 3D structure when immersed in solutions. During incubation in the swelling media, an osmotic pressure is generated across the hydrogel, resulting in water uptake (swelling) or water loss (shrinking) [26]. SBF was used as it mimics the composition of human plasma, and therefore, is an ideal medium for swelling tests, as conducted in [27]. PBS was used as its pH of 7.4 also closely matches the pH of the human body. The swelling results for the WPI-based biomaterials are presented in Figure 2A,B. When interpreting the box plots, a taller box plot shows a wider interquartile range, with the top of the box representing the upper quartile and the bottom of the box representing the lower quartile. Within the box plot, the line represents the median value and the minimum and maximum are shown by the whiskers.

Figure 2A shows clear trends for each sample group in PBS. The 40/0 and 40/2.32 biomaterials showed a decrease in swelling over time. The 40/0 biomaterials showed no significant change in the first 24 h, and then, began to decrease. The 40/2.32 biomaterials showed a small initial increase in the first 24 h before beginning to decrease. In contrast, the 40/5 biomaterials displayed an increase in swelling over time. The 40/5 biomaterials continued to swell over the recorded time intervals with a large increase in the first 24 h compared to the other groups. For 40/0 and 40/2.32, the swelling ratio results are precise, as shown by the short box plots. However, the measurements for the 40/5 biomaterials show large variance as the box plots are much taller, and therefore, less precise; hence, the true swelling ratio cannot be concluded. To determine the concentration at which the CaSiO_3_ swelling ratio changes from an overall decreasing trend to an increasing trend, one could form hydrogels of intermediate CaSiO_3_ concentrations between 2.32% and 5%.

Figure 2B also shows clear trends for each sample group in SBF. Similarly to the swelling results in PBS, the 40/0 and 40/2.32 biomaterials showed a decrease in swelling over time, whereas the 40/5 biomaterials showed an increase in swelling over time. This may be due to both solutions having a pH of 7.4. The 40/0 biomaterials showed the same trend in both SBF and PBS. The 40/2.32 biomaterials incubated in SBF showed a different trend to those incubated in PBS; in SBF, swelling initially decreased and continued to decrease over time, with a much lower swelling ratio at 168 h. The 40/5 biomaterials continued to swell over the recorded time intervals with a large increase in the first 24 h compared to the other groups. Similarly to the swelling ratio results obtained in PBS, the swelling ratio results are precise for the 40/0 and 40/2.32 biomaterials, as shown by the short box plots. However, this is not the case for the 40/5 biomaterials as the box plots are much taller, and therefore, imprecise, although the variance in the swelling ratios for the 40/5 biomaterials in SBF is smaller compared to that in PBS.

It was expected that the addition of CaSiO_3_ would decrease the swelling ratio due to an increased cross-linking density of the polymer matrix, resulting in less free space for the fluid to be absorbed. An example of this is the swelling data reported by Slota et al., who incorporated HA into a WPI hydrogel and incubated the resulting composite into SBF. It was suggested that the spaces between the polymer chains were filled due to the incorporation of the ceramic phase, preventing fluid sorption [28]. Also, it has been reported in the literature that a decrease in swelling can increase the material’s mechanical properties [29]. In natural bone repair, fractures take six to eight weeks to heal; therefore, the hydrogel-based biomaterial should not degrade too quickly within this period [30].

### 3.3. Mechanical Properties

**Figure 3:**
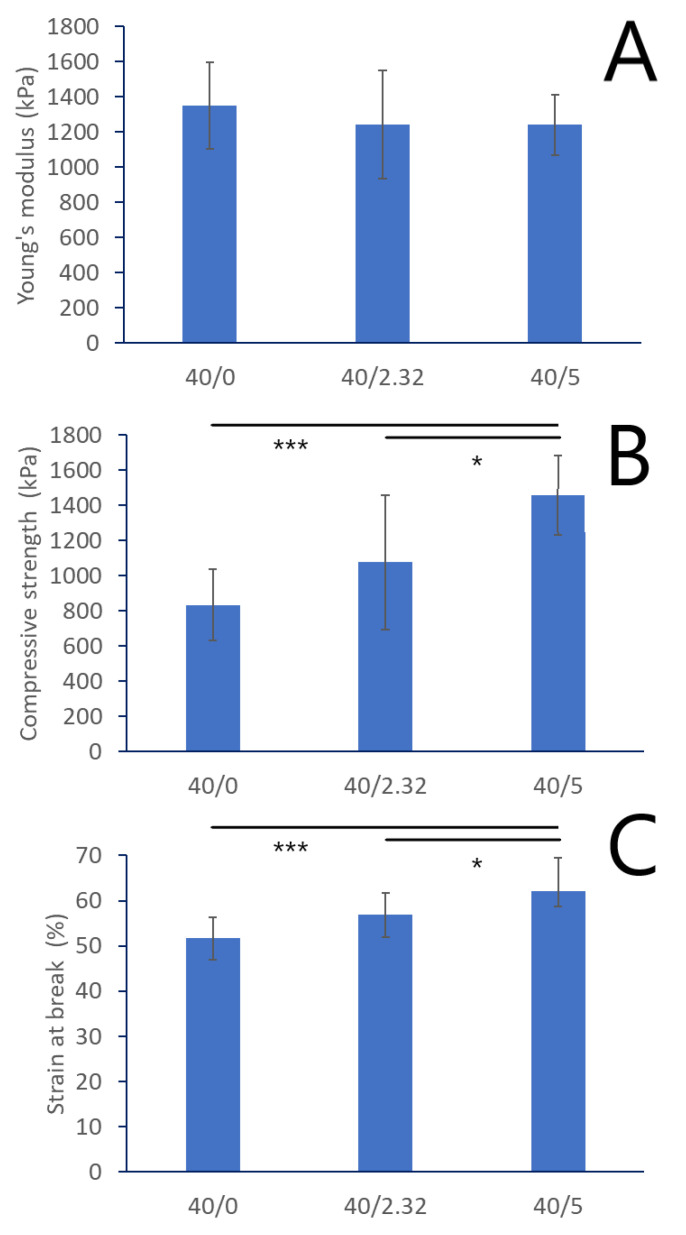
Comparative compression test results for WPI-based biomaterials: 40/0 (control), 40/2.23, and 40/5 (*n* = 10). (**A**) Young’s Modulus; (**B**) ultimate compressive strength; (**C**) compressive strain at break. Error bars show standard deviation. Significances based on One-Way ANOVA test followed by Tukey’s multiple comparison, *p* < 0.05 (*: *p* < 0.05; ***: *p* < 0.001).

Typical stress–strain curves for each biomaterial group are shown in Supplementary Figure S1. Young’s Modulus, ultimate compressive strength, and strain at break were determined; the results are shown in Figure 3A–C, respectively. The values are comparable to those shown in previous work by Dziadek et al. [26]. No significant differences were observed in Young’s Modulus values between the different biomaterial groups. Ultimate compressive strength and strain at break increased with increasing CaSiO_3_ content. However, the reasons for these increases remain unclear. The similarity of the values of Young’s Modulus suggests that the differences in swelling ability (Figure 2) are not due to differences in stiffness.

### 3.4. ATR-FTIR Results

**Figure 4:**
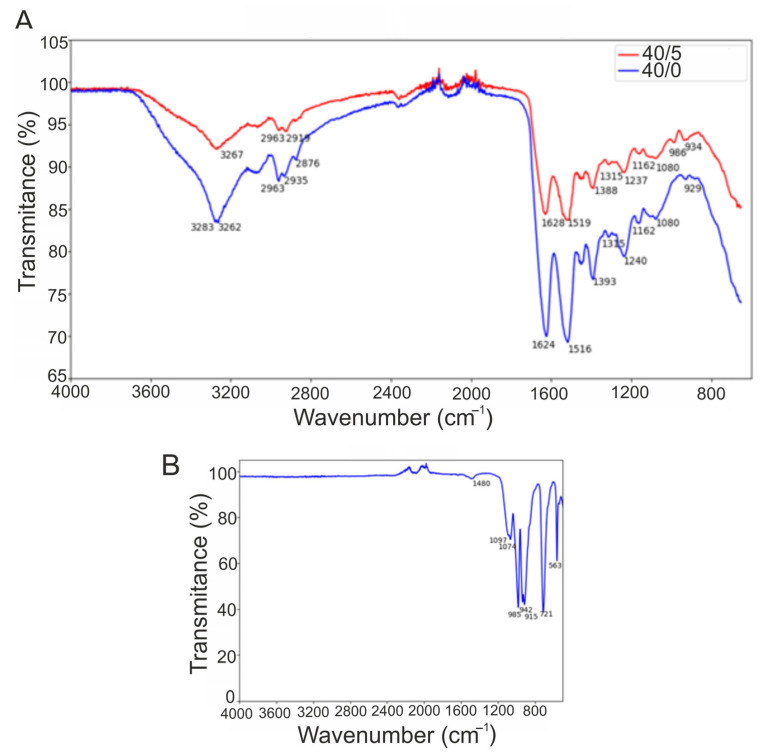
ATR-FTIR spectra for WPI-based biomaterials: 40/0 (control) and 40/5 (**A**) as well as CaSiO_3_ powder (**B**).

Based on data obtained from earlier experiments (Figure 1, Figure 2 and Figure 3), only 40/0 and 40/5 biomaterials were selected for further analysis. From the ATR-FTIR analysis, the functional groups of the biomaterial can be determined by analyzing the bonds present. As the main component of the biomaterial is the matrix formed of WPI, and as the main composition of whey protein consists of β-lactoglobulin, the expected bonds are those that correspond to peptide bonds within proteins, i.e., amide regions. Figure 4A shows the spectra for both the 40/0 and 40/5 biomaterials. When the exact wavenumber is not assigned, the closest wavenumber is used from the reference material for Table 3 and is shown in brackets. The reference material used is Movasaghi et al. [31]. Therefore, these peak assignments correspond to bonds found in natural tissue and organic materials. Consequently, some of the assignments for the 40/5 biomaterials in Table 3 will not be assigned correctly using this reference material as CaSiO_3_ is an inorganic material. However, it is not known which bonds correspond to the WPI matrix or the ceramic phase. Therefore, due to this, the CaSiO_3_ powder was analyzed using ATR-FTIR spectroscopy. Figure 4B shows the spectra obtained. Comprehensive reference material could not be found to fully characterize the graph; therefore, multiple sources were used. When wavenumbers were not exact, it could be due to the resolution of the equipment, but they were still included up to a maximum of 5 cm^−1^ difference. Nandiyanto et al. [20] quantified the frequency of the silicate ion as 1100–900 cm^−1^, which could correspond to the peak at 985 cm^−1^. Furthermore, Paluskiewicz et al. [32] investigated wollastonite (CaSiO_3_) and, using ATR-FTIR, assigned the 985 cm^−1^ peak to stretching non-bridging Si–O. Also, the bonds present in this powder at 1097 cm^−1^ and 1074 cm^−1^ were observed at 1092 cm^−1^ and 1072 cm^−1^, respectively, in their research, and these peaks were assigned to stretching bridging Si–O bonds. The peak at 563 cm^−1^ could be assigned to the Si-O flexural vibration usually seen at 567 cm^−1^ [33].

From Figure 4A and Table 3 a comparison between the two dried hydrogel biomaterials can be performed. Some identical peaks are present in both biomaterials, including CH_3_ modes, amide III, the stretching modes of the C-OH groups, and phosphate vibrations. However, there are also some shifts in band wavelengths, meaning that the bands have the same assignment but different band wavenumbers, including stretching O-H and C-H and amide I and II; this can be attributed to the 4 cm^−1^ resolution. These identical or slightly shifted peaks are observed at lower wavenumbers for the WPI control biomaterial (40/0). Lower transmittance means there is a high population of bonds that have vibrational energies corresponding to the incident light [34]. Comparing the results of the 40/0 spectra to those in the literature provided by Gbassi et al. [35], all the bonds found within the single-bond region (4000–2500 cm^−1^) and the double-bond region (2000–1500 cm^−1^) are observed to have only some small shifts in wavenumber. However, in the fingerprint region (1500–600 cm^−1^) some bonds were not observed in the 40/0 biomaterial. On the other hand, there are also bonds present in the 40/0 biomaterial which are absent from the example from the literature. No bonds are present in the triple-bond region (2500–2000 cm^−1^) either.

Using the assignments of the CaSiO_3_ and comparing the peaks to the 40/5 biomaterial suggests that the peaks at 985 cm^−1^ and 986 cm^−1^, respectively, show the presence of silicate ions. This is the only bond in the 40/5 spectra that is not present in the 40/0 spectra. As Si-O and Ca-O are the main bonds in CaSiO_3_, the presence of these bonds would be expected in the FTIR spectra. However, the bands that are caused by Si-O flexural vibration and Ca-O stretching vibration appear at low wavenumbers (567, 509, 472, and 453 cm^−1^) [33]. However, the spectra obtained for the dried hydrogel biomaterials were only collected in the range of 4000–600 cm^−1^. Hence, in future FTIR investigations, the wavenumber range should be extended to wavenumbers below 600 cm^−1^. This would allow for the detection of the aforementioned bonds.

### 3.5. Human Osteoblasts Response In Vitro

**Figure 5:**
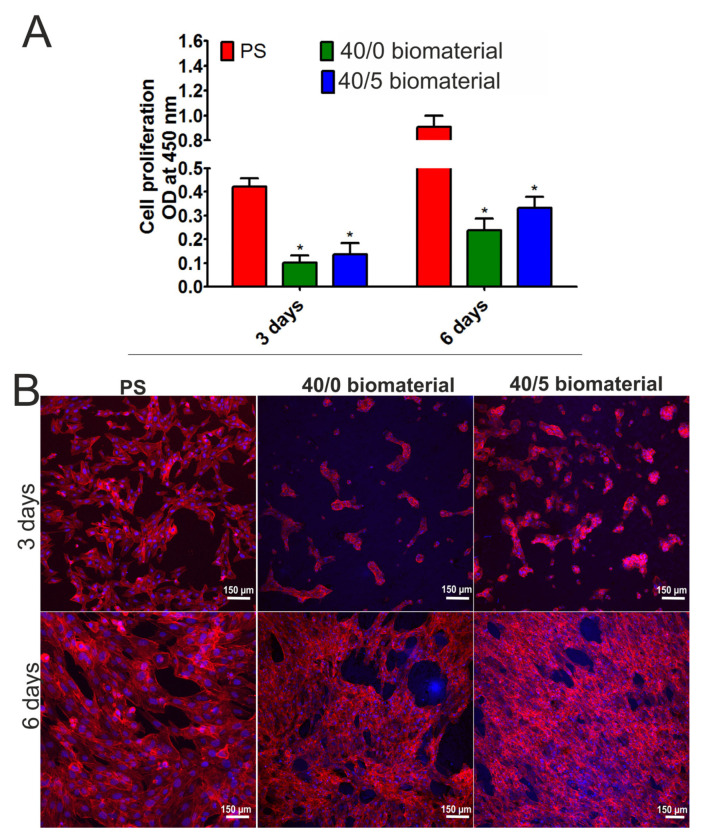
Proliferation of human osteoblasts cultured on the 40/0 and 40/5 biomaterials after 3 and 6 days of incubation. Metabolic activity was assessed via the WST-8 assay (**A**). * Significantly different results compared to control (cells cultured on polystyrene, PS) according to Two-Way ANOVA test, followed by Bonferroni comparison test, *p* < 0.05. No statistical differences were observed between the biomaterials. Cell morphology (**B**) was visualized by staining cell nuclei (Hoechst 33342 dye) and actin filaments of the cytoskeleton (AlexaFluor 635 dye). Cells were observed under a confocal microscope, magnification 100×, scale bar = 150 μm.

In the first stage of the cell experiments, the effect of biomaterials on the proliferation of human osteoblasts was assessed. The WST-8 assay enables the assessment of cell metabolic activity by measuring OD values. The OD value is directly proportional to the number of living, dividing cells and is therefore a measure of cell viability and proliferation. It was demonstrated that the metabolic activity of human osteoblasts cultured on both tested biomaterials (40/0 and 40/5) increased over time (Figure 5A). Importantly, it was noted that cells that grew on the 40/5 biomaterials had higher metabolic activity compared to cells maintained on the 40/0 hydrogels both after 3 and 6 days of incubation, but the differences were not statistically significant (*p* > 0.05). Observations using a confocal microscope (Figure 5B) confirmed the results obtained in the WST-8 assay. Osteoblasts that grew on both biomaterials showed normal morphology and their number increased with the lengthening of the experiment time. The number of cells growing on the 40/5 biomaterial was higher compared to the number of cells growing on the 40/0 biomaterial. Thus, these results indicated that both biomaterials supported the growth of viable, metabolically active osteoblasts, but the 40/5 biomaterial, which was enriched with CasiO_3_, enhanced cell proliferation more potently than the 40/0 biomaterial.

**Figure 6:**
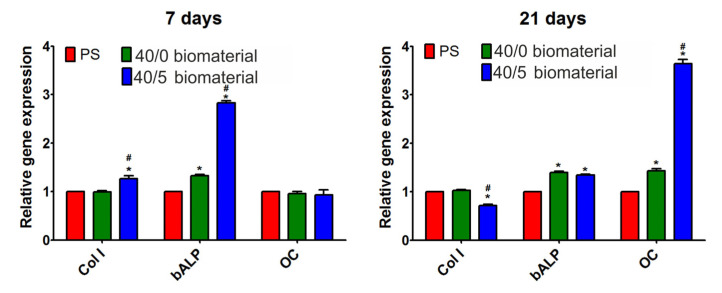
Relative expression level of genes: collagen I (Col I), bone alkaline phosphatase (bALP), and osteocalcin (OC) in human osteoblasts that grew on 40/0 and 40/5 biomaterials. The data were normalized to the expression level of genes in cells maintained on polystyrene (PS). * Significantly different results compared to expression level in cells that grew on PS; ^#^ Significantly different results compared to expression level in cells that grew on WPI hydrogel; One-Way ANOVA test followed by Tukey’s multiple comparison, *p* < 0.05.

The second stage of cell research was the assessment of the differentiation of human osteoblasts in direct contact with biomaterials. After 7 days of incubation, RT-qPCR analysis (Figure 6) demonstrated that osteoblasts that grew on the 40/0 biomaterials expressed a significantly higher amount of bALP compared to cells cultured on polystyrene (PS). In turn, the expression of collagen I and osteocalcin in these cells was at a similar level to that in cells that grew on PS. In the case of cells that grew on the 40/5 biomaterials, an increased expression of collagen I and bALP was observed both in comparison with the cells that grew on PS and with cells cultured on the 40/0 biomaterials. After 21 days of incubation, osteoblasts that grew on the 40/0 biomaterials showed increased expression of bALP and osteocalcin compared to cells cultured on PS. Similarly, cells cultured on the 40/5 biomaterials exhibited significantly higher expression of bALP and osteocalcin compared to cells that grew on polystyrene. Importantly, the expression of osteocalcin in cells cultured on the 40/5 biomaterials was almost three times higher compared to its expression in cells cultured on the 40/0 biomaterials. It is known that during osteoblast differentiation, the amounts of produced osteogenic markers (such as collagen I, bALP, osteocalcin) change over time. In the first phase of differentiation, osteoblasts produce a high amount of collagen I; then, in the second phase, the cells secrete large amounts of bALP; and finally, in the third phase, they mainly produce osteocalcin [21]. Therefore, it is clear that both biomaterials supported osteogenic differentiation. Nevertheless, taking into account the almost threefold higher expression of bALP after 7 days of incubation (second phase of differentiation) and the almost threefold higher expression of osteocalcin after 21 days of incubation (third phase of differentiation) in cells cultured on the 40/5 biomaterials compared to cells cultured on the 40/0 biomaterials, it should be emphasized that the addition of CaSiO_3_ to the WPI-based biomaterial significantly supported the process of osteoblast differentiation.

## 4. Conclusions

This study demonstrated the effect of the addition of CaSiO_3_ to a WPI-based hydrogel on its structural, physicochemical, mechanical, and biological properties in vitro. The results obtained permitted an initial estimation of the biomedical potential of the fabricated biomaterials as future bone scaffolds. It was demonstrated that the addition of CaSiO_3_ to WPI-based hydrogels resulted in an increase in the compressive strength of the biomaterials. Indeed, among the tested biomaterials, the best results were obtained for the biomaterial with the highest concentration of CaSiO_3_ (40/5). Our cell culture experiments showed that this biomaterial (40/5) exhibited very high cytocompatibility, as it promoted osteoblast proliferation and osteogenic differentiation in vitro.

Considering the aforementioned results, it seems that the WPI (40/5) biomaterial may be considered a promising candidate for bone tissue engineering applications. However, to evaluate its biomedical potential precisely, additional in vivo studies should be performed. In future, we plan to conduct animal studies to evaluate its safety and ability to promote the regeneration of bone defects. For these reasons, an evaluation of inter alia subchronic toxicity and the ability to undergo osseointegration will be performed.

## Figures and Tables

**Table 1:** The percentages of WPI and CaSiO_3_ in fabricated composite biomaterials.

Sample Symbol	Content of WPI (%)	Content of CaSiO_3_
40/0	40	0
40/2.32	40	2.32
40/5	40	5

**Table 2:** The primers used for RT-qPCR analysis. The primer’s sequences for Col I, bALP, and GAPDH were projected using the Primer-Blast tool [24]. The primer sequences for OC were developed based on data from the literature [25].

Gene	Primer Sequence (5′–3′)	Product Size (bp)
Collagen type I (Col I)	F: GGCCCAGAAGAACTGGTACA R: AATCCATCGGTCATGCTCTC	81
Bone alkaline phosphatase (bALP)	F: TTGGCCAACAGGGTAGATTT R: GGAGGGTCAGATCCAGAATG	144
Osteocalcin (OC)	F: ACACTCCTCGCCCTATTG R: GATGTGGTCAGCCAACTC	249
Glyceraldehyde-3-phosphate dehydrogenase (GAPDH)	F: CACCACACTGAATCTCCCCT R: TGGTTGAGCACAGGGTACTT	115

**Table 3:** The spectral interpretations of the 40/0 and 40/5 biomaterials from the FTIR spectra according to recommendations of Movasaghi et al. [31].

Wavenumber (cm^−1^)	Assignment
3283 (3273/87/89)	Symmetric stretching O-H
3267 (3273/87/89)	Symmetric stretching O-H
3262 (3273/87/89)	Symmetric stretching O-H
2963	Deformation CH_3_
2935	Asymmetric stretching C-H
2919 (2917/8/9)	Stretching C-H
2876 (2874)	Symmetric stretching CH_3_ Stretching C-H, N-H Symmetric stretching CH_3_ of acyl chains (lipids)
1628 (1630–700)	Amide I region
1624 (1630–700)	Amide I region
1519 (1517)	Amide II
1516 (1517)	Amide II
1393 (1935)	Bending CH_3_ due to aliphatic side groups of the amino acid residues
1388 (1388)	Bending CH_3_ Stretching C-O, deformation C-H, deformation N-H
1315 (1317)	Amide III band components of proteins
1240	Asymmetric stretching PO_2_^−^ Amide III mode of protein
1237	Asymmetric stretching PO_2_^−^ (phosphate I)
1162	Stretching modes of the C-OH groups of proteins, e.g., serine, threonine, and tyrosine residues
1080	Symmetric stretching PO_2_^−^
986 (985)	OCH_3_ modes
934 (938)	Unassigned
929 (925–9)	Unassigned

## Data Availability

Available upon request.

## Supplementary Materials

The following supporting information can be downloaded at: https://www.mdpi.com/article/10.3390/ma16196484/s1, Figure S1: Typical stress-strain curves obtained during compression testing for WPI-based biomaterials: 40/0 (control), 40/2.23, and 40/5.

## References

[1] Bai X., Gao M., Syed S., Zhuang J., Xu X., Zhang X.Q. (2018). Bioactive hydrogels for bone regeneration. Bioact. Mater..

[2] Liu J., Yang L., Liu K., Gao F. (2023). Hydrogel scaffolds in bone regeneration: Their promising roles in angiogenesis. Front. Pharmacol..

[3] Yue S., He H., Li B., Hou T. (2020). Hydrogel as a biomaterial for bone tissue engineering: A review. Nanomaterials.

[4] Shahshahani S., Shahgholi M., Karimipour A. (2023). The thermal performance and mechanical stability of methacrylic acid porous hydrogels in an aqueous medium at different initial temperatures and hydrogel volume fraction using the molecular dynamics simulation. J. Mol. Liq..

[5] Hafezi M., Khorasani S.N., Zare M., Neisiany R.E., Davoodi P. (2021). Advanced hydrogels for cartilage tissue engineering: Recent progress and future directions. Polymers.

[6] Madhusudanan P., Raju G., Shankarappa S. (2020). Hydrogel systems and their role in neural tissue engineering. J. R. Soc. Interface.

[7] Arjun Uppuluri V.N.V., Sathanantham S.T., Bhimavarapu S.K., Elumalai L. (2022). Polymeric Hydrogel Scaffolds: Skin Tissue Engineering and Regeneration. Adv. Pharm. Bull..

[8] Koochaki A., Shahgholi M., Sajadi S.M., Babadi E., Inc M. (2023). Investigation of the mechanical stability of polyethylene glycol hydrogel reinforced with cellulose nanofibrils for wound healing: Molecular dynamics simulation. Eng. Anal. Bound. Elem..

[9] Douglas T.E.L., Vandrovcová M., Kročilová N., Keppler J.K., Zárubová J., Skirtach A.G., Bačáková L. (2018). Application of whey protein isolate in bone regeneration: Effects on growth and osteogenic differentiation of bone-forming cells. J. Dairy Sci..

[10] Gupta D., Kocot M., Tryba A.M., Serafim A., Stancu I.C., Jaegermann Z., Pamuła E., Reilly G.C., Douglas T.E.L. (2020). Novel naturally derived whey protein isolate and aragonite biocomposite hydrogels have potential for bone regeneration. Mater. Des..

[11] Dziadek M., Kudlackova R., Zima A., Slosarczyk A., Ziabka M., Jelen P., Shkarina S., Cecilia A., Zuber M., Baumbach T. (2019). Novel multicomponent organic–inorganic WPI/gelatin/CaP hydrogel composites for bone tissue engineering. J. Biomed. Mater. Res. Part A.

[12] Tai C.S., Chen Y.Y., Chen W.L. (2016). β -Lactoglobulin Influences Human Immunity and Promotes Cell Proliferation. BioMed Res. Int..

[13] Srinath P., Abdul Azeem P., Venugopal Reddy K. (2020). Review on calcium silicate-based bioceramics in bone tissue engineering. Int. J. Appl. Ceram. Technol..

[14] Liu Z., He X., Chen S., Yu H. (2023). Advances in the use of calcium silicate-based materials in bone tissue engineering. Ceram. Int..

[15] Venkatraman S.K., Swamiappan S. (2020). Review on calcium- and magnesium-based silicates for bone tissue engineering applications. J. Biomed. Mater. Res. Part A.

[16] Youness R.A., Tag El-deen D.M., Taha M.A. (2023). A Review on Calcium Silicate Ceramics: Properties, Limitations, and Solutions for Their Use in Biomedical Applications. Silicon.

[17] Zhang N., Molenda J.A., Fournelle J.H., Murphy W.L., Sahai N. (2010). Effects of pseudowollastonite (CaSiO_3_) bioceramic on in vitro activity of human mesenchymal stem cells. Biomaterials.

[18] Klimek K., Przekora A., Benko A., Niemiec W., Blazewicz M., Ginalska G. (2017). The use of calcium ions instead of heat treatment for β-1,3-glucan gelation improves biocompatibility of the β-1,3-glucan/HA bone scaffold. Carbohydr. Polym..

[19] Kalaskar D., Zhang X. (2014). Inorganic Biomaterials: Structure, Properties and Applications.

[20] Nandiyanto A.B.D., Oktiani R., Ragadhita R. (2019). How to Read and Interpret FTIR Spectroscope of Organic Material. Indones. J. Sci. Technol..

[21] Klimek K., Palka K., Truszkiewicz W., Douglas T.E.L., Nurzynska A., Ginalska G. (2022). Could Curdlan/Whey Protein Isolate/Hydroxyapatite Biomaterials Be Considered as Promising Bone Scaffolds?—Fabrication, Characterization, and Evaluation of Cytocompatibility towards Osteoblast Cells In Vitro. Cells.

[22] Klimek K., Tarczynska M., Truszkiewicz W., Gaweda K., Douglas T., Ginalska G. (2022). Freeze-Dried Curdlan/Whey Protein Isolate-Based Biomaterial as Promising Scaffold for Matrix-Associated Autologous Chondrocyte Transplantation—A Pilot In-Vitro Study. Cells.

[23] Rao X., Huang X., Zhou Z., Lin X. (2013). An improvement of the 2ˆ(-delta delta CT) method for quantitative real-time polymerase chain reaction data analysis. Biostat. Bioinform. Biomath..

[24] Primer-BLAST Tool. http://www.ncbi.nlm.nih.gov/tools/primer-blast/.

[25] Hashemibeni B., Dehghani L., Sadeghi F., Esfandiari E., Gorbani M., Akhavan A., Tahani S.T., Bahramian H., Goharian V. (2016). Bone repair with differentiated osteoblasts from adipose-derived stem cells in hydroxyapatite/tricalcium phosphate in vivo. Int. J. Prev. Med..

[26] Dziadek M., Charuza K., Kudlackova R., Aveyard J., D’Sa R., Serafim A., Stancu I.C., Iovu H., Kerns J.G., Allinson S. (2021). Modification of heat-induced whey protein isolate hydrogel with highly bioactive glass particles results in promising biomaterial for bone tissue engineering. Mater. Des..

[27] Baino F., Yamaguchi S. (2020). The use of simulated body fluid (SBF) for assessing materials bioactivity in the context of tissue engineering: Review and challenges. Biomimetics.

[28] Slota D., Gląb M., Tyliszczak B., Dogulas T.E.L., Rudnicka K., Miernik K., Urbaniak M.M., Rusek-Wala P., Sobczak-upiec A. (2021). Composites based on hydroxyapatite and whey protein isolate for applications in bone regeneration. Materials.

[29] Killion J.A., Geever L.M., Devine D.M., Higginbotham C.L. (2014). Fabrication and in vitro biological evaluation of photopolymerisable hydroxyapatite hydrogel composites for bone regeneration. J. Biomater. Appl..

[30] Canillas M., De Lima G.G., Rodríguez M.A., Nugent M.J.D., Devine D.M. (2016). Bioactive composites fabricated by freezing-thawing method for bone regeneration applications. J. Polym. Sci. Part B Polym. Phys..

[31] Movasaghi Z., Rehman S., Rehman I.U. (2008). Fourier transform infrared (FTIR) spectroscopy of biological tissues. Appl. Spectrosc. Rev..

[32] Paluszkiewicz C., Blazewicz M., Podporska J., Gumuła T. (2008). Nucleation of hydroxyapatite layer on wollastonite material surface: FTIR studies. Vib. Spectrosc..

[33] Chen W., Liang Y., Hou X., Zhang J., Ding H., Sun S., Cao H. (2018). Mechanical grinding preparation and characterization of TiO_2_-coated wollastonite composite pigments. Materials.

[34] Satyanarayana R. (2020). Ground Characterization and Foundations Proceedings of Indian Geotechnical Conference.

[35] Gbassi G., Yolou F., Sarr S., Atheba P., Amin C., Ake M. (2012). Whey proteins analysis in aqueous medium and in artificial gastric and intestinal fluids. Int. J. Biol. Chem. Sci..

