# Supplementary material for: Whey Protein Isolate/Calcium Silicate Hydrogels for Bone Tissue Engineering Applications—Preliminary In Vitro Evaluation
Source: Materials (Basel). 2023 Sep 29;16(19):6484. doi: 10.3390/ma16196484 (PMC10573410; doi:10.3390/ma16196484)
Supplement: Supplementary file 1 [file materials-16-06484-s001.zip › materials-2626730-supplementary.pdf]

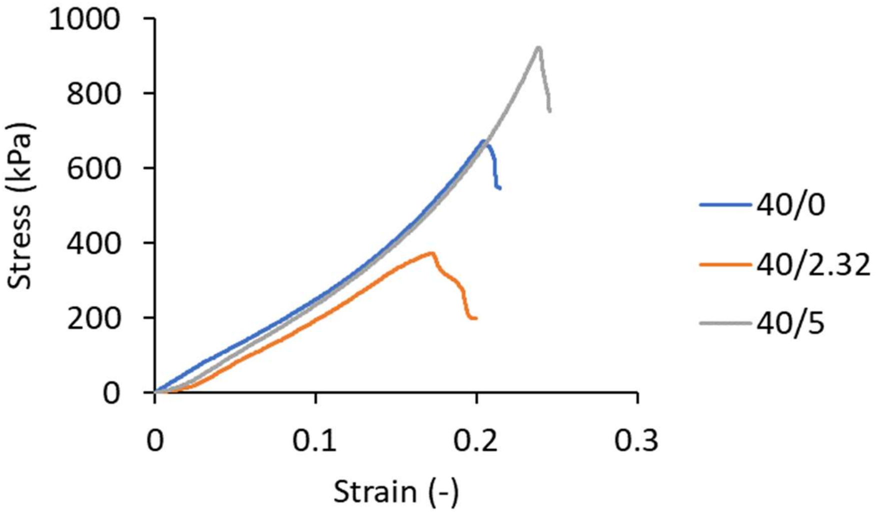

Supplementary Figure S1. Typical stress-strain curves obtained during compression testing for WPI-based biomaterials: 40/0 (control), 40/2.23, and 40/5.
